# Ceramide Regulates Anti-Tumor Mechanisms of Erianin in Androgen-Sensitive and Castration-Resistant Prostate Cancers

**DOI:** 10.3389/fonc.2021.738078

**Published:** 2021-09-17

**Authors:** I Gusti Md Gde Surya C. Trapika, Xin Tracy Liu, Long Hoa Chung, Felcia Lai, Chanlu Xie, Yang Zhao, Shaohui Cui, Jinbiao Chen, Collin Tran, Qian Wang, Shubiao Zhang, Anthony S. Don, George Qian Li, Jane R. Hanrahan, Yanfei Qi

**Affiliations:** ^1^Centenary Institute of Cancer Medicine and Cell Biology, University of Sydney, Camperdown, NSW, Australia; ^2^School of Pharmacy, Faculty of Health and Medicine, University of Sydney, Camperdown, NSW, Australia; ^3^Department of Endocrinology, Royal Prince Alfred Hospital, Sydney, NSW, Australia; ^4^Chinese Medicine Anti-Cancer Evaluation Program, Central Clinical School, University of Sydney, Camperdown, NSW, Australia; ^5^Department of Biochemistry and Molecular Biology, School of Medicine & Holistic Integrative Medicine, Nanjing University of Chinese Medicine, Nanjing, China; ^6^Key Laboratory of Biotechnology and Biorescources Utilization of Ministry of Education, Dalian Minzu University, Dalian, China; ^7^Translational Cancer Metabolism Laboratory, School of Medical Sciences and Prince of Wales Clinical School, UNSW, Sydney, NSW, Australia; ^8^School of Medical Sciences, Faculty of Health and Medicine, University of Sydney, Camperdown, NSW, Australia

**Keywords:** apoptosis, cell cycle arrest, autophagy, endoplasmic reticulum stress, sphingolipid

## Abstract

Prostate cancer is the second most prevalent malignancy worldwide. In the early stages, the development of prostate cancer is dependent on androgens. Over time with androgen deprivation therapy, 20% of prostate cancers progress to a castration-resistant form. Novel treatments for prostate cancers are still urgently needed. Erianin is a plant-derived bibenzyl compound. We report herein that erianin exhibits anti-tumor effects in androgen-sensitive and castration-resistant prostate cancer cells through different mechanisms. Erianin induces endoplasmic reticulum stress-associated apoptosis in androgen-sensitive prostate cancer cells. It also triggers pro-survival autophagic responses, as inhibition of autophagy predisposes to apoptosis. In contrast, erianin fails to induce apoptosis in castration-resistant prostate cancer cells. Instead, it results in cell cycle arrest at the M phase. Mechanistically, C16 ceramide dictates differential responses of androgen-sensitive and castration-resistant prostate cancer cells to erianin. Erianin elevates C16 ceramide level in androgen-sensitive but not castration-resistant prostate cancer cells. Overexpression of ceramide synthase 5 that specifically produces C16 ceramide enables erianin to induce apoptosis in castration-resistant prostate cancer cells. Our study provides both experimental evidence and mechanistic data showing that erianin is a potential treatment option for prostate cancers.

## Introduction

Prostate cancer (PCa) is the second most common cancer and the sixth highest cause of cancer-related mortality worldwide (1). In the United States, PCa accounts for 26% and 11% of all cancer incidence and death in men, respectively (2). In the early stages of development, PCa relies on androgen to stimulate its growth, and thus it is sensitive to androgen deprivation therapy (3, 4). However, 20% of patients receiving androgen deprivation therapy progress to castration-resistant PCa (CRPC) within 2-3 years. CRPC is associated with a significantly higher degree of drug resistance (5). Meanwhile, androgen deprivation therapy has shown adverse effects, including cardiovascular, metabolic and cognitive morbidities (4). Taxanes, such as docetaxel and cabazitaxel, are applied as primary chemotherapy in CRPC patients (3, 4, 6). These chemicals can disrupt microtubule dynamics during both mitosis and interphase, leading to cell cycle arrest and even death in PCa (6, 7). However, approximately half of patients treated with taxanes develop drug resistance (8). The prognosis and overall survival rate of PCa remain unsatisfactory, and searching for new treatment options is still the key matter in PCa research (6).

Natural products are valuable sources of both new anti-cancer medicines and lead compounds (9). Bibenzyls are a class of phenolic compounds generated as ethane derivatives from the flavonoid biosynthetic pathway in plants (10, 11). Among the 89 bibenzyls identified from the *Dendrobium* genus, 23 compounds exhibit anti-tumor properties by inducing apoptosis, inhibiting cell proliferation and suppressing cell migration and invasion in multiple cancer cell lines [summarized in (11)]. Erianin is a major bibenzyl compound extracted from *Dendrobium*. This phytochemical has little toxicity in immortalized normal human hepatocytes and nasopharyngeal normal primary cells (12, 13), whereas it has shown potent anti-tumor effects at nanomolar concentrations. Erianin induces oxidative stress, leading to apoptosis *via* the intrinsic apoptosis pathway (14–18), blocks the cell cycle at the G2/M phase (12, 14, 17), and has been recently found to trigger ferroptosis, an iron-dependent programmed cell death (19). In addition, treatment of nude mice with erianin significantly inhibit the growth of lung cancer, osteosarcoma, colorectal cancer and bladder cancer xenografts (14, 16, 17, 19–21). However, the anti-tumor effects of erianin in PCa have not been examined.

Endoplasmic reticulum (ER) stress-associated apoptosis is one of the primary targets of anti-cancer drug discovery (22), and a large number of bioactive natural products elicit anti-tumor activities in this manner (23, 24). The ER is an intracellular membrane network, maintaining proteostasis within the cell. Perturbance of ER homeostasis will activate unfolded protein responses (UPRs) through three canonical signaling axes, including PKR-like ER kinase (PERK)-eukaryotic initiation factor 2α (eIF2α), inositol-requiring enzyme 1α (IRE1α), and activating transcription factor 6 (ATF6), leading to adaptation to the stress (22, 25). In response to severe and prolonged stress, all three signaling arms activate pro-apoptotic UPRs, converging at the induction of C/EBP homologous protein (CHOP) (25). CHOP transcriptionally upregulates Bim, a pro-apoptotic Bcl-2 family member, which is believed to be the mechanistic link between ER stress and the intrinsic apoptosis pathway (26). Erianin induces phosphorylation of c-Jun N-terminal kinases (JNK), a signaling node associated with both ER stress and oxidative stress (14, 16, 18). However, a definitive answer to whether erianin provokes ER stress-associated apoptosis still remains elusive.

Autophagy is another adaptive response of cells to cope with stress (27). Upon the activation of autophagy, pro-light chain 3 (LC3) is cleaved in the cytosol to form LC3-I, followed by the lipidation of LC3-I to yield LC3-II (28). LC3-II is localized to autophagosomes and drives their maturation (28). After the fusion of autophagosomes and lysosomes, cargoes contained in autophagosomes, such as p62, are degraded along with LC3-II in lysosomes (29). Highly dependent on the context, autophagy can be either pro-survival or pro-death, as a double-edged sword. In addition, chemotherapies often induce autophagy in parallel with apoptosis (30, 31). Thus, although autophagy is a popular target in anti-cancer clinical trials, care must be taken to understand the role of autophagy in the context of the specific disease state (32). Erianin has been demonstrated to induce pro-survival autophagy in human osteosarcoma cells (14), making it intriguing to examine the role of erianin-induced autophagy in PCa.

Sphingolipids are a class of essential lipids, functioning as both membrane constituents and signaling molecules within the cell (33). Metabolically, sphingolipids are interconnected in a network with ceramide as the central hub (33). Ceramide can be biosynthesized from the condensation of amino acids and fatty acids, followed by reduction, acylation and desaturation (33). Ceramide can be degraded *via* the sphingolipid catabolic pathway into non-lipid products (33). In ceramide biosynthesis, ceramide synthases (CerS) mediate the rate-limiting step that adds the fatty acyl chain to the sphingoid base (34). There are six mammalian isoforms of CerS, designated as CerS1-6 (34). Of them, CerS5 and CerS6 predominantly produce C16 ceramide (35). In the prostate, CerS5 is more prevalent than CerS6 (36). Ceramide orchestrates apoptosis, ER stress and autophagy signaling (37, 38). However, different ceramide species may exhibit distinct biological roles. For example, C16 ceramide is a well-established pro-apoptotic signal, whereas C24 ceramide is often anti-apoptotic (39–41). In addition, C16 ceramide has different biological effects at spatially distinct subcellular compartments. For instance, ablation of CerS6 but not CerS5 confers metabolic protection in the liver, as CerS6 mainly produces C16 ceramide in mitochondria (42). So far, research on the role of ceramide in prostate cancer has not reached the level of the subcellular lipid pool or lipid subtype. Instead, total ceramide levels have been found to determine therapy resistance in PCa cells. The anti-cancer drug camptothecin elevates total ceramide mass and thus induces apoptosis in androgen-sensitive LNCaP PCa cells, but it fails to do so in castration-resistant PC3 PCa cells (43). Accumulation of ceramide by blocking its catabolism potentiates the apoptosis of PC3 cells (44, 45). However, whether erianin regulates ceramide remains untested.

In this study, we assessed the anti-tumor properties of erianin, including its effects on clonogenicity, cell migration and cell viability in LNCaP and PC3 cells. To elucidate the anti-tumor pathways induced by erianin, we examined ER stress-associated apoptosis and cell cycle arrest. Here we identified that erianin elicits discrepant anti-tumor mechanisms in LNCaP and PC3 cells. We also examined whether erianin regulated ceramide levels in PCa cells and elucidated whether C16 ceramide determines PCa cell susceptibility to erianin-induced apoptosis. Collectively, our study aims to demonstrate the potential therapeutic benefits of erianin against PCa and elucidates the underlying anti-tumor mechanism.

## Material and Methods

### Cell Culture and Treatments

LNCaP and PC3 PCa cells, original from American Type Culture Collection (ATCC), were gifted by Dr. Mu Yao and Prof. Thomas Grewal at the University of Sydney, Australia, respectively. The cell cultures were maintained at 37 °C in a humidified incubator with 5% CO_2_. All PCa cell lines were cultured in phenol red-free Roswell Park Memorial Institute (RPMI) 1640 medium (Thermo Fisher) containing 10% fetal bovine serum and 1% penicillin/streptomycin. Erianin, bafilomycin A1 and S-trityl-L-cysteine (STLC) were purchased from Sigma, while z-VAD was obtained from BioVision. All treatments were dissolved in dimethyl sulfoxide (DMSO). PC3 cells were transfected with CerS5 and CerS6 plasmids (Genscript) using lipofectamine LTX Plus reagents (Thermo Fisher).

### Colony Formation Assay

LNCaP or PC3 cells were seeded at 1,000 or 500 cells/well in 6-well plates, respectively. Cells were allowed to stabilize for 24 h before the treatment with erianin for an additional ten days. Colonies were then fixed with 4% cold paraformaldehyde (Thermo Fisher) for 15 min, followed by the crystal violet (0.5% w/v, Sigma) staining for another 15 min at room temperature. Images were captured using a ChemiDoc™ Touch Imaging System (Bio-Rad laboratories) and processed using ImageLab™ Software (Bio-Rad Laboratories).

### Wound-Healing Assay

The wound-healing assay was performed to examine cell migration of LNCaP and PC3 cells, as described previously (46). In brief, cells were seeded at 10^5^/ml in 96 well plates, allowing them to grow to 70-80% of confluency. Cells were scratched using Wound Maker 96™ (Essen BioScience). The media containing cell debris were discarded. Wound closure was imaged using an Incucyte S3 (Essen BioScience) at multiple time points up to 48 h after erianin treatment. Relative cell migration was calculated using Incucyte S3 software.

### Cell Viability Assay

Cell viability was determined by 3-(4,5-dimethyl-thiazol-2-yl)-5-(3-carboxymethoxyphenyl)-2-(4-sulfophenyl)-2H-tetrazolium, inner salt (MTS) assay using the colorimetric CellTiter^®^ 96 AQueous Cell Proliferation Assay kit (Promega) (47). Cells were cultured in 96 well plates. At the time of measurement, 20 μl of MTS solution (containing phenazine methosulfate) was added to 100 μl of normal growth medium. Cells were incubated with the reagents for 1 h at 37 °C humidified incubator. The luminescence was determined at 490 nm on a TECAN Infinite M1000Pro plate reader.

### Flow Cytometry

Cell cycle was determined in cells fixed with 70% cold ethanol at 4 °C overnight. Ethanol was added dropwise. The fixed cells were then washed with Dulbecco’s Phosphate Buffered Saline (DPBS, Thermo Fisher) and incubated with FxCycle™ PI/RNase Staining Solution (Thermo Fisher) at room temperature in the dark for 30 minutes. Samples were analyzed using BD LSRFortessa™ X-20 (BD™ Biosciences). For the apoptosis assays, living cells were co-stained with propidium iodide (PI) and annexin V-FITC (Thermo Fisher) and analyzed on a BD LSR-II (BD™ Biosciences) (48).

### Western Blotting

Proteins were extracted with cell lysis buffer containing 1% Triton X-100, sonicated using QSonica-Q800R2, quantified using bicinchoninic acid assay (Sigma) and separated on NuPAGE™ 4-12% Bis-Tris precast gels (Thermo Fisher). Immunoblot analyses were conducted according to standard protocol with the following antisera: caspase-3 (#9662), PARP (#9542), β-actin (#3770), IRE1α (#3294), p-eIF2α (#3597), t-eIF2α (#2103), p-P38 MAPK (#4511), t-P38 MAPK (#8690), p-JNK1/2 (#4668), t-JNK1/2 (#9252), p-ERK1/2 (#9101), t-ERK1/2 (#9102), CHOP (#5554), Bim (#2933), Bcl-2 (#4223), Bax (#5023), p-Akt (#4060), t-Akt (#9272), GAPDH (#5174), cyclin b1 (#12231), p-CDK1 substrates (#9477) and FLAG (#14793) from Cell Signaling Technology; p62 (#ab56416) and Mcl-1 (#ab31948) antibodies were purchased from Abcam, while LC3 antibody (#NB100-2220) was obtained from Novus Biologicals. Images were captured using a ChemiDoc™ Touch Imaging System and processed using ImageLab™ Software.

### Confocal Microscopy

Using lipofectamine LTX plus reagents (Thermo Fisher), cells were transfected with GFP-LC3 plasmid, gifted by Prof. Pu Xia, Fudan University, China. LC3 puncta were visualized under treatments. Immunofluorescent staining of γ-tubulin was performed in cells fixed with 4% paraformaldehyde, permeabilized in 2% Triton X-100, blocked in 3% fatty acid-free bovine serum albumin (BSA), incubated with fluorophore-bound γ-tubulin antibody (Thermo Fisher, #MA1-850-A555) and counterstained in ProLong™ Gold Antifade Mountant with DAPI. Confocal microscopy was carried out using a Nikon C2 microscope.

### Lipidomics

Ceramides, sphingosine and S1P were analyzed as described in our previous work (49). In brief, these sphingolipids were quantified on a Thermo Fisher TSQ Altis triple quadrupole mass spectrometer, operated in positive ion mode, coupled to a Vanquish UHPLC system (Thermo Fisher). Lipids were separated on an Agilent Eclipse Plus C8 column. Peaks were integrated using Xcalibur software (Thermo Fischer). Individual sphingolipid species were normalized to class-specific internal standards (Avanti Polar Lipids).

### Statistics

Comparisons between two groups were analyzed by unpaired two-tailed *t*-tests, and multiple comparisons were analyzed by ANOVA with Tukey tests, using GraphPad Prism 9. Differences at values of *P* < 0.05 were considered significant.

## Results

### Anti-Tumor Properties of Erianin in PCa

**Figure 1:**
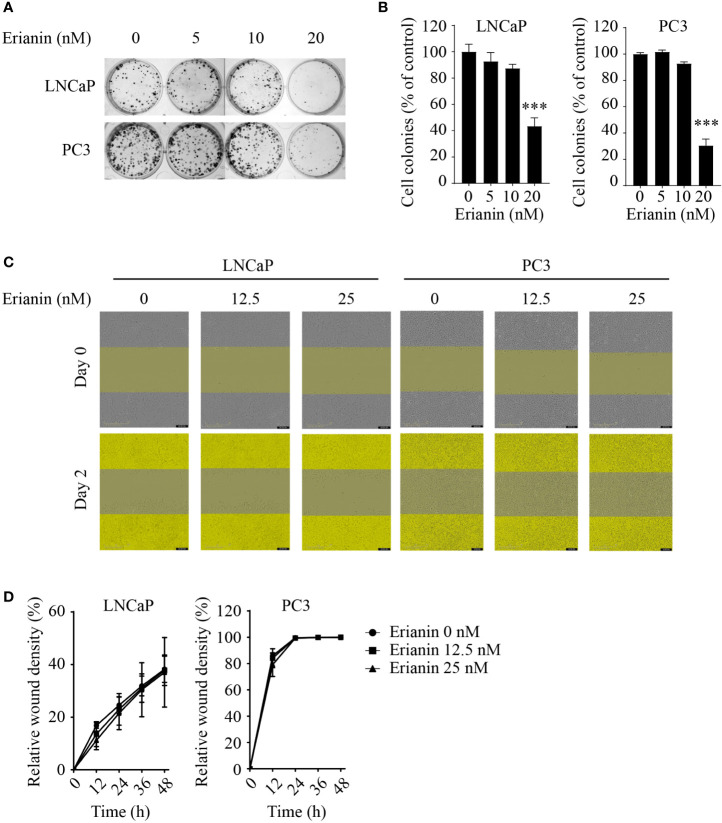
Erianin suppresses colony formation but not migration in both LNCaP and PC3 cells. **(A, B)** Cells were treated with erianin at indicated concentrations for 10 days. Images are representative of three independent experiments **(A)** and the number of colonies was quantified **(B)**. **(C, D)** Wound healing assay was performed to assess cell migration. Cells were treated with erianin at indicated concentrations for up to 48 h. Images are representative of three independent experiments **(C)** and the wound closure area were quantified **(D)**. Data are expressed as mean ± SD. n=3. ***p < 0.001, *versus* untreated control.

To investigate the anti-tumor effects of erianin in PCa, we first examined the clonogenic survival of LNCaP and PC3 cells upon erianin treatment (**Figures 1A, B**). Erianin at 20 nM inhibited cell colony formation by 56.5% and 69.6% in LNCaP and PC3 cells, respectively. We also examined whether erianin suppressed PCa cell migration by performing the wound healing assay (**Figures 1C, D**), following previous erianin studies in other cancer types (17, 19, 20). However, erianin at 12.5 and 25 nM failed to alter the relative wound density in the scratched areas compared with untreated control during 48 h. Therefore, erianin did not affect PCa cell migration at concentrations sufficient to significantly suppress clonogenicity.

### Erianin Induces ER Stress-Associated Apoptosis in LNCaP Cells

**Figure 2:**
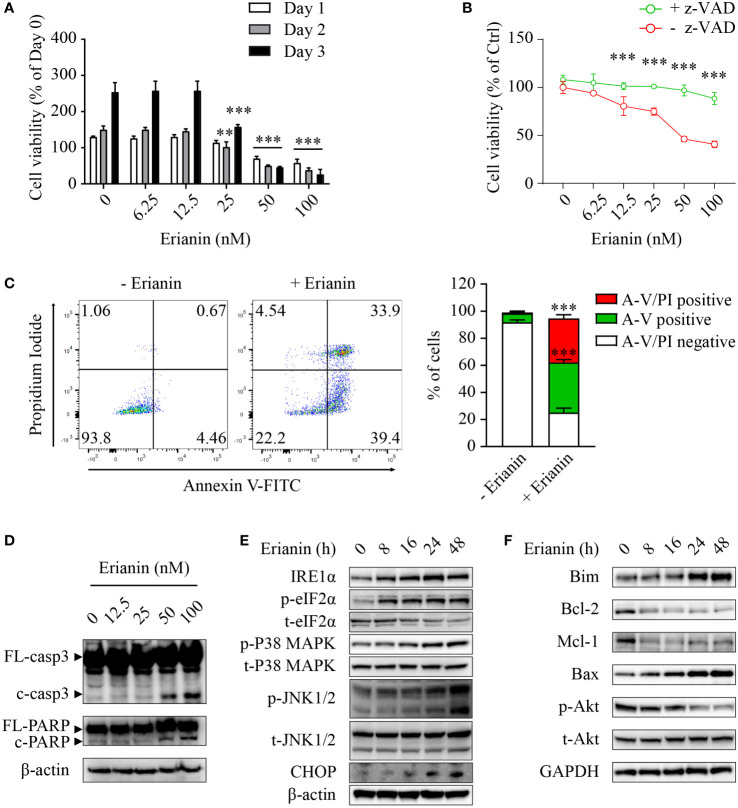
Erianin induces ER stress-associated apoptosis in LNCaP cells. **(A, B)** LNCaP cell viability was determined by MTS colorimetric assay. LNCaP cells were treated with erianin at indicated concentrations for 1, 2 and 3 days **(A)**. LNCaP cells were treated with erianin at indicated concentrations in the presence or absence of pan-caspase inhibitor z-VAD at 50 µM for 24 h **(B)**. Data are shown as a percentage of untreated control at day 0. **(C)** LNCaP cells were treated with erianin at 100 nM for 24 h and apoptosis was analyzed by flow cytometry with propidium iodide (PI) and annexin V-FITC (A-V) co-staining. A-V single positive population represents early apoptotic cells, while A-V and PI double-positive population refers to late apoptotic/dead cells. **(D–F)** LNCap cells were treated with erianin at indicated concentrations for 24 h **(D)** or at 50 nM for indicated times **(E, F)**. Apoptosis **(D)**, ER stress **(E)** and Bcl-2 family members **(F)** were examined by Western blotting. FL-, full length; c-, cleaved; casp3, caspase 3. Data are expressed as mean ± SD. n=3. **p < 0.01; ***p < 0.001, *versus* untreated control.

Having examined the anti-tumor effects of erianin, we explored if erianin induced apoptosis in LNCaP cells. We found that erianin decreased LNCaP cell viability in a dose-dependent manner (**Figure 2A**). The IC_50_ of erianin was 33.8 nM, 26.5 nM and 26.2 nM at 24 h, 48 h and 72 h, respectively. Co-treatment with the pan-caspase inhibitor z-VAD profoundly suppressed the erianin-induced decrease in cell viability, indicating that erianin induced apoptosis in LNCaP cells (**Figure 2B**). To further define the pro-apoptotic effect of erianin, we performed flow cytometry using annexin V-FITC and PI staining (**Figure 2C**). Treatment with erianin at 100 nM led to early apoptosis and late apoptosis/death in 37.0% and 32.5% of LNCaP cells, respectively (**Figure 2C**). Erianin-induced apoptosis was also confirmed by the cleavage of caspase-3 and poly(ADP-ribose) polymerase (PARP), classic markers of apoptosis (**Figure 2D**). We next investigated how erianin induced apoptosis in LNCaP cells. Treatment with erianin induced activation of JNK1/2 and expression of CHOP, both pro-apoptotic UPRs (**Figure 2E**). This resulted from the activation of upstream UPRs, including upregulation of IRE1α and phosphorylation of eIF2α at earlier time points (**Figure 2E**). In parallel, phosphorylation of p38 MAPK was also induced by erianin (**Figure 2E**). In line with this, erianin decreased protein expression levels of anti-apoptotic Bcl-2 family members, such as Bcl-2 and myeloid cell leukemia 1 (Mcl-1), whereas it increased levels of pro-apoptotic Bcl-2 family members, like Bim and Bax (**Figure 2F**). In addition, erianin repressed the phosphorylation of Akt, a well-studied oncogene, contributing to programmed cell death (**Figure 2F**). These data indicated that erianin induced apoptosis by triggering pro-death ER stress responses.

### Inhibition of Autophagy Potentiates Erianin-Induced Apoptosis in LNCaP Cells

**Figure 3:**
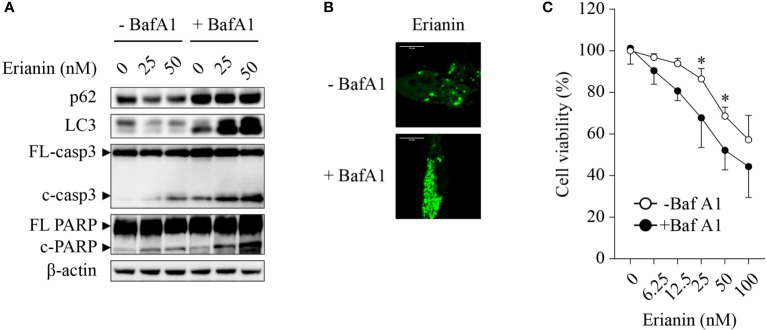
Erianin induces pro-survival autophagy in LNCaP cells. **(A)** LNCaP cells were treated with erianin at indicated concentrations for 24 h in the presence or absence of 100 nM bafilomycin A1 (BafA1, an autophagy inhibitor). Levels of p62, LC3, cleaved caspase-3 and cleaved PARP were examined by Western blotting. FL-, full length; c-, cleaved; casp3, caspase 3. **(B)** LNCaP cells were transfected with GFP-tagged LC3 for 48 h. Then cells were co-treated with 100 nM BafA1 and 50 nM erianin for 24 h. Confocal images were captured. Bar = 10 µm. **(C)** Cells were treated with erianin at indicated concentrations in the presence or absence of 100 nM bafilomycin A1 for 24 h. Cell viability was determined by MTS colorimetric assay. Data are expressed as mean ± SD. n=4. *p < 0.05, -BafA1 *versus* +BafA1.

Chemotherapies often induce autophagy, either pro-survival or pro-death. Thus, we interrogated whether erianin induced autophagy in LNCaP cells and, if so, what the role of erianin-induced autophagy was in cell death. Treatment with erianin resulted in a decreased level of LC-3 (**Figure 3A**). However, the reduced level of LC-3 could be derived from an accelerated autophagic flux or blockade of autophagic initiation. To clarify the effects of erianin on autophagy, we treated LNCaP cells with the lysosome inhibitor bafilomycin A1 that blocked the degradation of autophagosomes in lysosomes. In the presence of bafilomycin A1, treatment with erianin caused an enhanced accumulation of LC-3, indicating that erianin promoted autophagy (**Figure 3A**). Confirmed by confocal microscopy, erianin increased the aggregation of GFP-LC3 puncta, a typical feature of autophagosome formation during autophagy, in cells co-treated with bafilomycin A1 (**Figure 3B**). In addition, inhibition of autophagy by bafilomycin A1 potentiated erianin-induced apoptosis signaling (**Figure 3A**) and cell death (**Figure 3C**), indicative of a prosurvival role of erianin-induced autophagy.

### Erianin Fails to Induce Apoptosis in PC3 Cells

**Figure 4:**
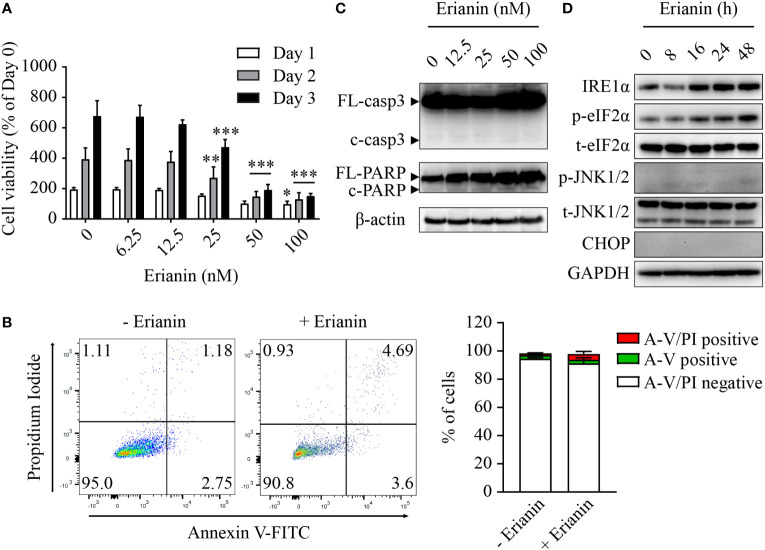
Erianin fails to induce apoptosis in PC3 cells. **(A)** PC3 cell viability was determined by MTS colorimetric assay in cells treated with erianin at indicated concentrations for 1, 2 and 3 days. Data are shown as a percentage of untreated control at day 0. n=4. **(B)** Cells were treated with erianin at 100 nM for 24 h and apoptosis was analyzed by flow cytometry with propidium iodide (PI) and annexin V-FITC (A-V) co-staining. A-V single positive population represents early apoptotic cells, while A-V and PI double-positive population refers to late apoptotic/dead cells. n=3. **(C, D)** Cells were treated with erianin at indicated concentrations for 24 h **(C)** or at 100 nM for indicated times **(D)**. Apoptosis and ER stress were examined by Western blotting. FL-, full length; c-, cleaved; casp3, caspase 3. Data are expressed as mean ± SD. *p < 0.05; **p < 0.01; ***p < 0.001, *versus* untreated control.

Similar to LNCaP cells (**Figure 2A**), treatment with erianin significantly decreased the number of viable PC3 cells, with IC50 values between 26-28 nM over 24-72 h (**Figure 4A**). However, we found differences between these two seemingly similar results. In LNCaP cells, erianin at 50 and 100 nM for 72 h reduced viable cell number to a level below the initial value (< 50% of day 0), suggestive of cell death (**Figure 2A**); whereas in PC3 cells treated with erianin at the same doses for 72 h, viable cell number was profoundly lowered as compared with the untreated control, but it was still 150% of the cell number on day 0, indicative of cell growth inhibition rather than cell death (**Figure 4A**). To confirm that erianin suppressed cell proliferation but did not induce cell death in PC3 cells, we performed apoptosis assays by flow cytometry with annexin V-FITC/PI staining and Western blotting for cleaved caspase-3 and PARP. Erianin at 100 nM did not induce significant early apoptosis or late apoptosis/death in PC3 cells (**Figure 4B**). Meanwhile, no cleaved caspase-3 or PARP was observed in immunoblots (**Figure 4C**). We also examined UPRs in erianin-treated PC3 cells. Erianin induced adaptive UPRs, e.g., IRE1α upregulation and eIF2α phosphorylation, whereas it failed to trigger pro-death UPRs, such as JNK1/2 phosphorylation and CHOP expression (**Figure 4D**). All these data indicate that erianin inhibited cell proliferation but did not induce cell death in PC3 cells.

### Erianin Induces Cell Cycle Arrest in PC3 Cells

**Figure 5:**
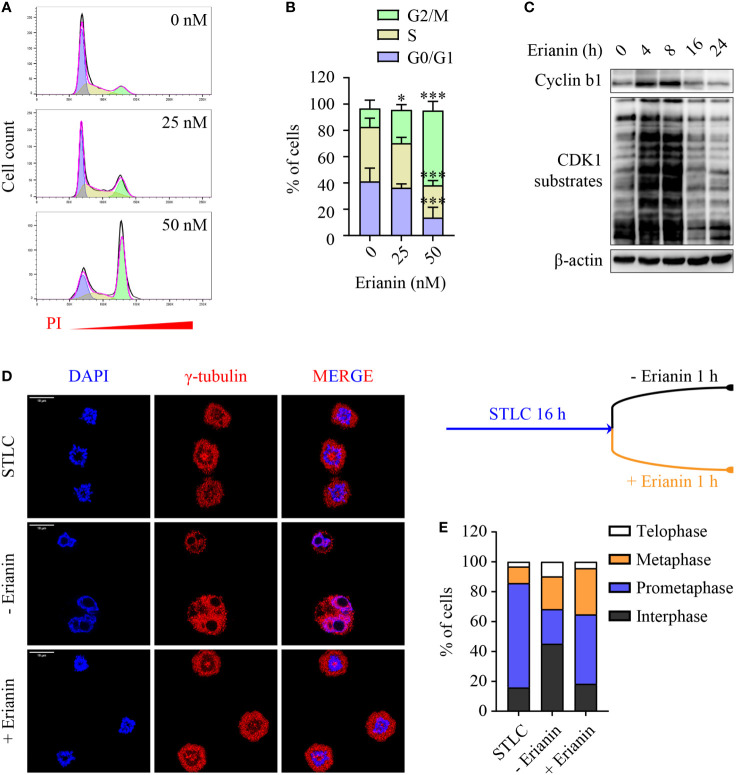
Erianin induces cell cycle arrest at the M phase in PC3 cells. **(A, B)** PC3 cells were treated with erianin at indicated concentrations for 24 h. **(A)** Cell cycle phase was determined by flow cytometry with propidium iodide (PI) staining. G_0_/G_1_, S and G_2_/M phase populations were estimated using Flowjo software and are indicated in purple, yellow and green, respectively. **(B)** Percentages of cells at each phase was quantified by Flowjo software. Data are expressed as mean ± SD. n=5. *p < 0.05; ***p < 0.001, *versus* untreated control. **(C)** PC3 cells were treated with erianin at 50 nM for indicated times. Cyclin b1 and phosphorylated CDK1 substrates were examined by Western blotting. **(D, E)** Cells were treated with S-trityl-L-cysteine (STLC) at 10 µM for 16 h to synchronize cells at the prometaphase. Then STLC was withdrawn and cell cycle progression to metaphase, telophase and interphase were monitored in the presence ot absence of erianin at 100 nM for 1 h. Confocal images were captured. Bar = 10 µm **(D)**. Percentages of cells at each cell cycle phase were quantified **(E)**.

Having demonstrated the anti-proliferative effects of erianin, we further examined how it affected the cell cycle in PC3 cells. We found that erianin induced a prominent cell cycle arrest at the G2/M phase (**Figures 5A, B**), consistent with significantly reduced cell proliferation (**Figure 4A**). Interestingly, erianin induced a transient upregulation of cyclin b1 and activation of CDK1 (**Figure 5C**), both essential for G2-to-M transition, suggesting that erianin blocked the cell cycle at the M phase. To confirm this, we synchronized cells at the prometaphase using STLC (50), followed by STLC washout and exposure to erianin for 1 h (**Figure 5D**). After 16 h treatment with STLC, 70% of cells were arrested at the prometaphase (**Figure 5E**). In the presence of erianin, there were still approximately 50% of cells unable to progress to metaphase even after STLC-mediated blockade was eased (**Figure 5E**). In contrast, only 23% of control cells failed to progress through the prometaphase (**Figure 5E**). Therefore, erianin exhibited anti-tumor effects in PC3 cells mainly *via* inhibition of mitosis.

### Erianin Fails to Induce Apoptosis in PC3 Cells Due to a Low Level of C16 Ceramide

**Figure 6:**
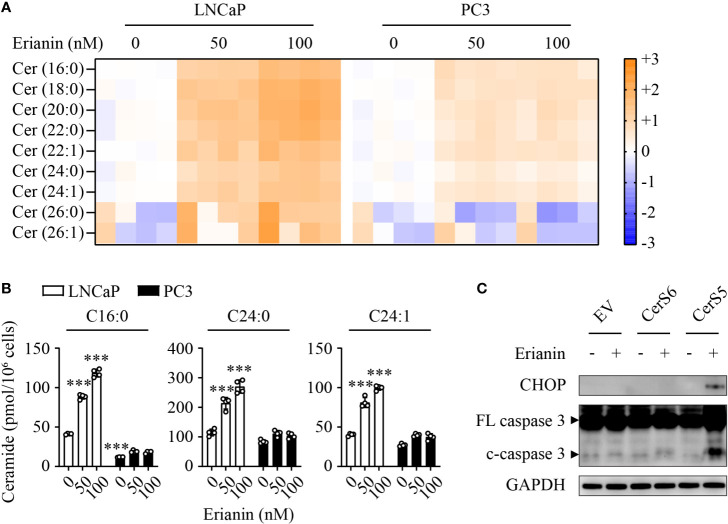
C16 ceramide determines PCa cell response to erianin. **(A, B)** LNCaP and PC3 cells were treated with erianin at indicated concentrations for 24 h. Lipids were extracted and analyzed using targeted lipidomics. Lipid heatmap shows the changes in levels of ceramides (Cer), sphingosine (Sph) and sphingosine 1-phosphate (S1P). The color key indicates the fold change on a log2 scale **(A)**. Absolute levels of C16:0, C24:0 and C24:1 ceramides **(B)**. Data are expressed as mean ± SD. n=4. ***, *p*<0.001, *versus* untreated LNCaP cells. **(C)** PC3 cells were transfected with empty vector (EV), ceramide synthase 5 (CerS5) and CerS6 for 48 h, prior to the treatment with erianin at 100 nM for an additional 24 h. CHOP and cleaved caspase-3 were examined by Western blotting.

To understand why LNCaP and PC3 cells responded differentially to erianin, we performed targeted sphingolipidomics to examine intracellular ceramide levels. Erianin profoundly elevated ceramide levels in a dose-dependent manner in LNCaP cells, whereas the change in ceramide levels was marginal in PC3 cells (**Figure 6A**). In both cell types, the three most abundant ceramide species were C16:0, C24:0 and C24:1. Interestingly, the basal (untreated) C16 ceramide level was significantly lower in PC3 cells as compared with LNCaP cells (11.7 *vs* 41.2 pmol/10^6^ cells, **Figure 6B**). When cells were treated with 100 nM erianin, this difference was further increased by 6.6 fold (**Figure 6B**). To examine whether erianin failed to induce apoptosis in PC3 cells due to a low level of intracellular C16 ceramide, we overexpressed C16 ceramide-producing enzymes CerS5 and CerS6. As shown in **Figure 6C**, erianin induced both CHOP expression and caspase-3 cleavage when CerS5 was overexpressed in PC3 cells, indicating that C16 ceramide was a major determinant of PCa cell susceptibility to erianin-induced apoptosis. In contrast, overexpression of CerS6 failed to promote erianin-induced apoptosis in PC3 cells, suggesting C16 ceramide exhibited pro-apoptotic effects in a specific subcellular pool.

## Discussion

**Figure 7:**
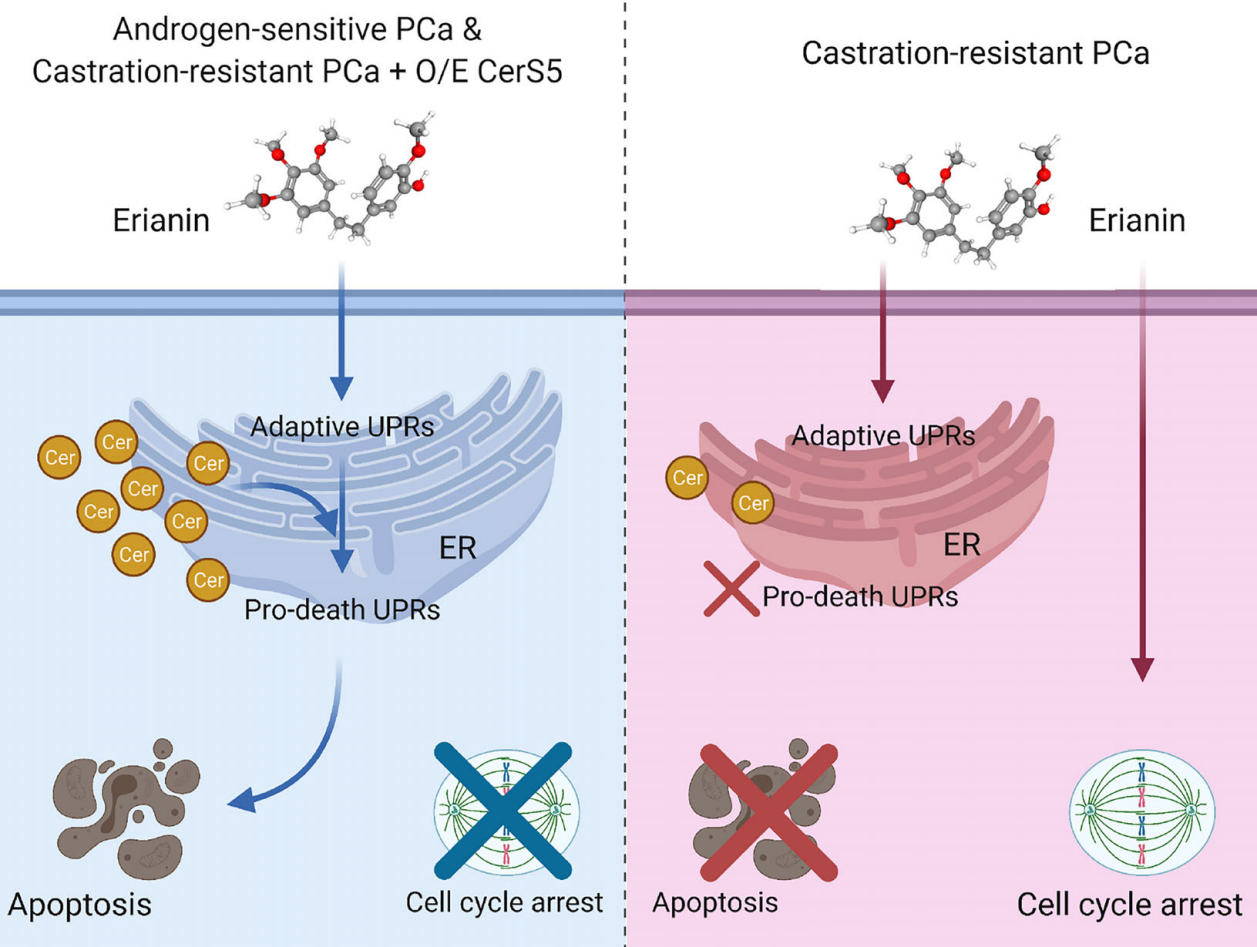
Model depicting discrepant anti-tumor effects of erianin in androgen-sensitive and castration-resistant prostate cancer (PCa) cells, in which intracellular level of ceramide plays a critical role. Left panel: Androgen-sensitive PCa cells and ceramide synthase 5 (CerS5)-overexpressed castration-resistant PCa cells exhibit a high level of intracellular ceramide. Under this condition, erianin induces both adaptive and pro-death unfolded protein responses (UPRs) at the endoplasmic reticulum (ER), leading to apoptosis. Right panel: Castration-resistant PCa cells exhibit a low level of intracellular ceramide. Under this condition, erianin can only induce adaptive UPRs but not pro-death UPRs or apoptosis. Alternatively, erianin induces cell cycle arrest at the M phase. The diagram was created with BioRender.com. The 3D conformer of erianin was adapted from PubChem, https://pubchem.ncbi.nlm.nih.gov/compound/Erianin#section=3D-Conformer, PubChem CID 356759.

In the present study, we have described the anti-tumor effects of erianin, a plant-derived natural compound, in both androgen-sensitive LNCaP and castration-resistant PC3 PCa cells. Erianin profoundly inhibited colony formation and cell viability of both cell types. We further identified that erianin exerted its anti-tumor activities *via* distinct mechanisms: a) In LNCaP cells, it activated multiple early and late UPRs, indicative of ER stress, which was associated with upregulation of pro-apoptotic Bcl-2 family members, Bim and Bax, and downregulation of anti-apoptotic Bcl-2 and its sibling protein Mcl-1, leading to the execution of apoptosis as reflected by cleavage of caspase-3 and PARP. Meanwhile, inhibition of autophagy sensitized LNCaP cells to erianin-induced apoptosis. b) In PC3 cells, erianin failed to induce pro-death UPRs and apoptosis. Instead, it inhibited cell proliferation by blocking mitosis but not the transition of the G2 to M phase. This is attributable to a low level of C16 ceramide in PC3 cells, as overexpression of C16 ceramide-producing enzymes CerS5 enabled erianin to trigger apoptosis signaling. Thus, this study has, for the first time, provided both functional and mechanistic evidence depicting the anti-tumor properties of erianin in PCa. We proposed a working model for the anti-tumor effects of erianin in PCa (**Figure 7**).

We chose LNCaP and PC3 cells in this study for three reasons: first, they are the most commonly used cell lines in PCa research, derived from a metastatic lesion of human prostatic adenocarcinoma and bone metastasis of grade IV PCa, respectively (51, 52). LNCaP cells express androgen receptor and prostate-specific antigen, whereas PC3 cells do not, and thus they recapitulate features of androgen-sensitive and castration-resistant PCa, respectively (53); second, a few studies demonstrate that tumor suppressor p53 determines PCa cell sensitivity to chemotherapies (54–56). Compared with LNCaP cells that express wild-type p53, PC3 cells are p53-null, and thus PC3 cells are usually more resistant to apoptosis; last, the anti-cancer drug camptothecin elevates total ceramide mass and induces apoptosis in LNCaP cells, but it fails to do so in PC3 cells (43). Therefore, these two cell lines are in marked contrast to examine the role of ceramide in erianin-induced apoptosis.

Erianin at nanomolar level inhibited colony formation in both LNCaP and PC3 cells (**Figures 1A, B**), which is consistent with previous reports in other cancer types (12, 14, 17, 19, 20). However, in contrast to the reported inhibitory effects on cancer cell migration (17, 19, 20), erianin at 12.5 and 25 nM failed to suppress cell migration in both PCa cell lines (**Figures 1C, D**). Higher doses of erianin, 50 and 100 nM, caused cell death in LNCaP cells (**Figure 2A**) and proliferation arrest (**Figure 4A**) in PC3 cells, and thus these treatment conditions were not suitable for assessing the effects on cell migration. Meanwhile, erianin induced apoptosis in LNCaP cells (**Figure 2**), in line with its pro-apoptotic effects in other cancer types (14–17). However, it did not induce apoptosis in PC3 cells (**Figure 4**). In line with this, paclitaxel can induce apoptosis only in LNCaP but not PC3 cells (57). Knockdown of p53 blocks apoptosis signaling in androgen-sensitive PCa cells (56); whereas reexpression of wild-type p53 sensitizes p53-mutant or p53-null castration-resistant PCa cells to apoptosis (54–56).

ER stress-associated apoptosis is an important therapeutic target of natural products in cancers (23, 24). In response to ER stress stimuli, cells initiate self-recovery UPRs *via* three canonical pathways PERK-eIF2α, IRE1α and ATF6 (22, 25). When coping with severe insults, stressed cells choose to activate ER stress-mediated apoptosis as a protective mechanism for the whole organism (22, 25). The most established pro-apoptotic UPR is CHOP that can be activated by all three arms of UPRs (22, 25). As a transcription factor, CHOP regulates Bim and Bcl-2 expression, leading to the activation of mitochondria-dependent apoptosis (26). In addition to CHOP, JNK activation is also regarded as a crucial signaling node in ER-associated apoptosis. JNK activation is dependent on IRE1α, one of the three primary ER stress sensors (58), and it further transduces signals to the intrinsic apoptosis machinery by regulating Bcl-2 and Bim (59). We found that erianin induced IRE1α upregulation and eIF2α phosphorylation in both LNCaP and PC3 cells, indicating that it perturbed ER homeostasis and resulted in ER stress responses (**Figures 2E**, **4D**). Furthermore, erianin induced pro-apoptotic UPRs, including JNK activation and CHOP expression, in LNCaP but not PC3 cells (**Figures 2E**, **4D**), which was associated with the execution of apoptosis (cleavage of caspase-3 and PARP) in respective cell types. In line with this, erianin-induced JNK activation has been demonstrated as an outcome of oxidative stress in previous studies (14, 16, 18), and elevated levels of PERK, IRE1α, ATF6 and CHOP have been reported in a study on erianin-loaded nanoparticles (60). Here, we identified that in addition to targeting mitochondria and the nucleus, erianin also induced apoptosis *via* ER stress, which provides a new avenue to the anti-tumor mechanism of this phytochemical.

In mammalian cells, the Bcl-2 family plays a central role in the regulation of apoptosis. Functionally and structurally, Bcl-2 family members are categorized into three groups, pro-apoptotic BH3-only proteins (e.g., Bim), anti-apoptotic proteins (e.g., Bcl-2 and Mcl-1) and pro-apoptotic effector proteins (e.g., Bax) (61). Anti-apoptotic Bcl-2 family members are often highly upregulated, while pro-apoptotic proteins are downregulated in cancers (62). Thus, Bcl-2 family members have been a panel of attractive targets for anti-cancer drug discovery over the past two decades (63). We found that erianin increased Bim and Bax levels whereas decreased Bcl-2 and Mcl-1 levels, which were associated with UPRs upstream and apoptosis signals downstream in LNCaP cells (**Figure 2F**). In support of this, previous studies also identify that erianin upregulates Bim and Bax expression whereas downregulates Bcl-2 and Mcl-1 levels, leading to the activation of the intrinsic apoptosis signaling in hepatocellular carcinoma cells, bladder cancer cells, nasopharyngeal cancer cells and osteosarcoma cells (13, 14, 16, 17). In addition, the Bcl-2 family has multiple functional interactions with Akt (protein kinase B), an oncogene that is highly expressed in almost all cancers. Akt elevates Bcl-2 expression *via* transcriptional regulation (64) and inhibits Bax translocation to mitochondria (65). Akt also promotes cell survival by repressing the transcription of Bim (66); however, Bim, in turn, can also dephosphorylate/inactivate Akt (67). We observed that erianin dephosphorylated Akt at S473, along with the changes of Bcl-2 family members (**Figure 2F**).

Furthermore, the Bcl-2 family also regulates autophagy. Bcl-2 and Mcl-1 inhibit autophagy through an inhibitory interaction with Beclin-1, an essential factor of autophagosome formation (68, 69). Reducing levels of anti-apoptotic proteins Bcl-2 and Mcl-1 result in dissociation of beclin-1, leading to activation of autophagy (70). Conversely, pro-apoptotic Bim also binds to beclin-1 and inhibits autophagosome formation (71). The autophagy-inducing effects of erianin have been observed in only a few studies (14, 72). In LNCaP cells, erianin increased Bcl-2 and Mcl-1 levels, believed to inhibit autophagy, whereas it decreased Bim levels, anticipated to activate autophagy (**Figure 2F**). Thus, it is intriguing to examine if erianin induces autophagy. As shown in **Figure 3A**, erianin decreased levels of LC3 and p62 in the absence of bafilomycin A1, suggestive of either an increased autophagic flux or a repressed autophagy initiation. Considering that p62, the cargo of autophagic degradation, was also decreased, erianin was likely to accelerate autophagic flux. To confirm this, we treated cells with bafilomycin A1 to retain LC3 by blocking its lysosomal degradation. When bafilomycin A1 inhibited autophagic degradation, LC3 was accumulated, as shown by Western blotting (**Figure 3A**) and confocal microscopy (**Figure 3B**), indicating that erianin enhanced autophagy in LNCaP cells. Autophagy can be either pro-survival or pro-death. We further clarified this by assessing the pro-apoptotic effects of erianin when autophagy was inhibited. Inhibition of autophagy profoundly sensitized LNCaP cells to erianin-induced apoptosis, indicating a prosurvival role of autophagy in this process (**Figures 3A, C**). Prosurvival autophagy has also been found in human osteosarcoma cells (14). Therefore, drug resistance derived from prosurvival autophagy should be considered in considering the implications of erianin for cancer therapy.

In difficult-to-kill cancer cells, halting their growth is an important anti-cancer strategy. Many phytochemicals induce cell cycle arrest in cancers with minimal toxicity and are considered an alternative approach in cancer treatment (73). In previous studies, erianin has been shown to induce cell cycle arrest at the G2/M phase, which is associated with apoptosis (12, 14, 16, 19). We also found that treatment with erianin caused a significant increase in the G2/M proportion of PC3 cells, where apoptosis was absent (**Figures 4**, **5A, B**). In marked contrast, erianin induced apoptosis in LNCaP cells (**Figure 2**), with no impact on their cell cycle (data not shown). Having demonstrated that erianin induced G2/M arrest, we further interrogated if cells failed to enter the M phase or encountered aberrant mitosis. There have been only two studies attempting to address this question, which drew inconsistent conclusions in different cell types (12, 14). We found that erianin increased expression levels of cyclin b1 and phospho-CDK1 substrates (**Figure 5C**), which are essential factors driving the G2-to-M transition (74, 75). This is aligned with the previous report that erianin induces DNA damage and mitotic catastrophe, which helps cells to overcome the G2-to-M checkpoint but rest at the M phase in hepatocellular carcinoma cells (12). We further elucidated that erianin withheld cells at prometaphase during the mitosis (**Figures 5D, E**).

Cell cycle arrest can trigger apoptosis signaling in cancers (76). However, the cell cycle arrest and apoptosis are disconnected in PC3 cells treated with erianin. This might be due to defective apoptosis signaling in this cell type, as PC3 cells lack p53 and express high levels of anti-apoptotic Bcl-2 family members (54–57, 77, 78). In addition, the intracellular level of ceramide is also implicated in the regulation of PCa cell sensitivity to chemotherapies (43). Increasing ceramide level inhibits clonogenic potential, tumorigenesis and lung metastasis of PC3 cells in a xenograft model (45). Multiple lines of evidence indicate that p53 and Bcl-2 family members regulate ceramide levels. As a transcription factor, p53 directly regulates the expression of CerS6, a ceramide synthase predominantly generating pro-apoptotic C16 ceramide (79). p53 also promotes the transcription of neutral sphingomyelinase, a key ceramide-producing enzyme (80). In contrast, p53 represses the expression of sphingosine kinase 1, a dominant enzyme mediating ceramide catabolism (81). In addition, treatment with either oxaliplatin or 5-fluorouracil dramatically increases CerS5 transcription in HCT-116 colon cancer cells, but this effect is abrogated when p53 is ablated (82), indicating that p53 is likely an upstream regulator of CerS5. Further, overexpression of anti-apoptotic Bcl-2 or Bcl-XL reduces ceramide production (83). These links between p53, Bcl-2 family and ceramide, taken together with their respective roles in apoptosis, place ceramide central to drug resistance in PC3 cells (37, 38). Indeed, we found that the level of C16 ceramide was significantly lower in PC3 cells than LNCaP cells (**Figures 6A, B**). Ceramide levels were increased under erianin treatment in LNCaP cells, whereas in PC3 erianin did not induce ceramide accumulation (**Figures 6A, B**). Remarkably, overexpression of the C16 ceramide-producing enzyme CerS5 but not CerS6 enabled erianin to induce apoptosis in PC3 cells (**Figure 6C**). The discrepant effects of CerS5 and CerS6 may be attributable to their tissue distribution (CerS5 is the predominant C16 ceramide-producing enzyme in the prostate) and subcellular localization (CerS6 is more specific to mitochondria) (36, 42). It is noted that C16 ceramides that are produced by CerS5 and CerS6 even have different subsets of binding proteins (42). Exactly why overexpression of CerS5 but not CerS6 sensitized PC3 cells to erianin-induced apoptosis warrants further investigation. In addition, pharmacological treatments that specifically increase C16 ceramide levels are worthy of testing in synergy with chemotherapies against castration-resistant PCa.

In summary, this study has provided both experimental and mechanistic data elucidating the anti-tumor properties of erianin in PCa cells. Erianin is a bibenzyl compound contained in *Dendrobium* species, predominantly *Dendrobium chrysotoxum*. We found that when PCa cells (LNCaP) possess intact death machinery, erianin induced apoptosis, whereas when cells (PC3) have defective apoptosis signaling, erianin caused the mitotic arrest. It is promising that erianin achieved these anti-tumor effects at the nanomolar level. Mechanistically, we also found ER stress as a new cause of erianin-mediated apoptosis. More importantly, we identified C16 ceramide as a determinant of drug resistance. Restoring C16 ceramide by the genetic approach enabled erianin to induce apoptosis in “difficult-to-kill” PC3 cells. In the past two years, growing evidence indicates the anti-tumor properties of erianin *in vivo*. Treatment of nude mice with erianin significantly suppresses the xenograft growth of H460 lung cancer cells in the right flank and lung parenchyma (100 mg/kg) (19), 143B osteosarcoma cells in the tibia (2 mg/kg) (14), SW480 colorectal cancer (50 mg/kg) (20), EJ bladder cancer cells in the right flank (50 mg/kg) (16). Although erianin at 100 mg/kg was found to induce haemorrhagic necrosis in BALB/c nude mice bearing Bel-7402 and A375 xenograft tumors (84), a more recent report shows that erianin at the same dose daily for 15 days significantly suppresses the growth of H460 lung cancer xenograft with no notable toxicity in normal tissues of lung, liver, kidney or spleen of BALB/c nude mice (19). However, compared with the high potency of erianin *in vitro*, the doses for *in vivo* studies are generally high. A further modification of the compound might be required to improve its bioavailability. In addition, more preclinical tests of erianin, combined with frontline PCa chemotherapy or in primary PCa mouse models, should be carried out with attention to any unwanted toxicity in the future.

## Data Availability Statement

The raw data supporting the conclusions of this article will be made available by the authors, without undue reservation.

## Author Contributions

YQ and JH conceived and coordinated the project. GL, AD, SZ, and QW contributed to project design. IT and XL performed the majority of experiments and data analyses. LC, FL, CX, YZ, SC, JC, and CT also contributed to data acquisition. YQ, LC and AD conducted lipidomic analyses. YQ and JH wrote the manuscript. All authors contributed to the article and approved the submitted version.

## Funding

This study was supported by National Health and Medical Research Council (Australia) Project Grant APP1162545 to YQ and Centenary Institute Future Leader Fellowship to YQ.

## Conflict of Interest

The authors declare that the research was conducted in the absence of any commercial or financial relationships that could be construed as a potential conflict of interest.

## Publisher’s Note

All claims expressed in this article are solely those of the authors and do not necessarily represent those of their affiliated organizations, or those of the publisher, the editors and the reviewers. Any product that may be evaluated in this article, or claim that may be made by its manufacturer, is not guaranteed or endorsed by the publisher.
